# Circulating cell-free nucleosomes as biomarkers for early detection of colorectal cancer

**DOI:** 10.18632/oncotarget.21908

**Published:** 2017-10-20

**Authors:** Louise Rasmussen, Ib Jarle Christensen, Marielle Herzog, Jake Micallef, Hans Jørgen Nielsen, For the Danish Collaborative Group on Early Detection of Colorectal Cancer

**Affiliations:** ^1^ Department of Surgical Gastroenterology 360, Hvidovre Hospital, Hvidovre, Denmark; ^2^ Belgian Volition SPRL, Isnes, Belgium; ^3^ Institute of Clinical Medicine, University of Copenhagen, Copenhagen, Denmark; ^4^ Appendix

**Keywords:** biomarkers, tumor, colorectal neoplasms, early detection of cancer, histone code

## Abstract

The aim was to evaluate serum levels of circulating cell-free nucleosomes (ccfn) containing a variety of epigenetic signals including 5-methylcytosine DNA, histone modifications H3K9Me3, H3K9Ac, H3S10PO4, H3K36Me3, H4K20Me3, H4PanAc and pH2AX, nucleosome variant H2AZ and nucleosome adducts with HMGB1 and EZH2 as well as ccfn per se, in addition to develop and evaluate predictor models based on the above mentioned ccfn and including serum levels of carcinoembryonic antigen (CEA), in early detection of colorectal cancer (CRC).

Blood-samples were collected from 4,105 individuals undergoing colonoscopy. Serum levels of ccfn and CEA were determined using enzyme-linked immunosorbent assays platforms. Individual assessment of levels of ccfn showed area under the receiver operating characteristic curve (AUCROC) = 0.525–0.576 in discrimination of individuals with CRC from individuals with non-malignant findings. Predictor models including ccfn containing 5-methylcytosine DNA, CEA, age and gender improved results (AUCROC = 0.736, sensitivity = 0.37 at specificity = 0.90). Further improvement was achieved in discrimination of individuals with CRC from individuals with clean colorectum (AUCROC = 0.840, sensitivity = 0.57 at specificity = 0.90). The levels of ccfn among patients with CRC appeared to be stage-independent. In conclusion, the performance of the developed predictor models is potentially promising in early detection of CRC.

## INTRODUCTION

During the last decade, major progress has emerged in diagnostics and treatment of colorectal cancer (CRC), but the disease still poses a major challenge to public health. CRC is one of the most frequent types of cancer and is estimated to account for some 700,000 deaths per year worldwide [1].

To counter this challenge, national population-based screening programs for CRC have been implemented in several countries, including Denmark [2]. Screening has been shown to reduce incidence and improve overall survival by early detection of CRC and by primary prevention through removal of pre-cancerous lesions (adenomas) [3, 4].

Colonoscopy is considered the Gold Standard in screening for CRC due to high diagnostic accuracy and the possibility of intervention (polypectomy, biopsy, etc.) during the examination. However, the feasibility of a colonoscopy-based screening program is challenged by capacity requirements, costs and side-effects, such as abdominal pain, transient cardiovascular changes, bleeding episodes and bowel perforation [5]. Therefore, fecal occult blood tests (FOBT) are often used to identify high-risk individuals, who should be offered subsequent diagnostic colonoscopy. At present, the most frequently applied FOBT in national screening programs is fecal immunochemical test (FIT) [2]; the sensitivity of FIT for detection of CRC is 0.79 at a specificity of 0.94 [6].

Despite the widespread use of feces-based tests in screening for CRC, the tests are challenged by low compliance; compliance for FIT is around 60% [7, 8] leading to a diminished effectiveness of screening. Hence, blood-based biomarkers, as an alternative or supplement to feces-based screening tests, have been subject to intense research. Blood samples are easy to retrieve, and the acceptance within a screening population consisting of mostly healthy individuals are higher compared with fecal samples required for FIT [9, 10]. Blood-based biomarkers are a heterogeneous group of various proteins, epigenetic markers, transcriptomes, metabolomes and circulating tumor-derived DNAs, and some have shown promising association to early stages of CRC [11–14]. In particular combination of various biomarkers have shown complimentary performance in discrimination of CRC [15–17], and the application of data fusion to incorporate information regarding age, gender, health-status, etc. to the determination of biomarkers has further increased the performance of some biomarkers [18].

Recent studies indicate that changes in levels of circulating cell-free nucleosomes (ccfn) may have potential as biomarkers for CRC [12, 19]. Nucleosomes consist of small DNA chains of approximately 147 bp, wrapped around a histone octamer that contains pairs of H2A, H2B, H3 and H4 proteins. Nucleosomes are bound together with linker DNA, linker histones and other non-histone proteins to form intracellular chromatin. During cell death the linker DNA is digested and nucleosomes are released into the circulation as ccfn, which can be detected by enzyme-linked immunosorbent assays (ELISA) [12]. Changes in levels of total ccfn have been shown in the circulation of individuals with cancer as a result of increased cell turn-over [20]. Furthermore, altered epigenetic control of gene expression (DNA methylation, modifications in histone structure, etc.) plays a crucial role in carcinogenesis [21]. Changes in levels of ccfn that contain CRC-specific epigenetic alterations can be detected in individuals with CRC [12, 19], and possess the potential to be a valuable biomarker in early detection of CRC.

The aim of the present study was to assess serum levels of ccfn containing 5-methylcytosine DNA (5 mC), histone H3 tri-methylated at lysine 9 (H3K9Me3), histone H3 acetylated at lysine 9 (H3K9Ac), histone H3 phosphorylated at serine 10 (H3S10PO4), histone H3 tri-methylated at lysine 36 (H3K36Me3), histone H4 tri-methylated at lysine 20 (H4K20Me3), pan-acetylated H4 (H4PanAc), histone variant H2AX phosphorylated at serine 139 (pH2AX), nucleosome variant H2AZ (H2AZ), nucleosome adducted with HMGB1 (HMGB1) and EZH2 (EZH2) as well as ccfn per se. In addition, development of predictor models based on serum levels of the above mentioned ccfn and including serum levels of carcinoembryonic antigen (CEA), age and gender were evaluated in early detection of CRC.

## RESULTS

**Figure 1:**
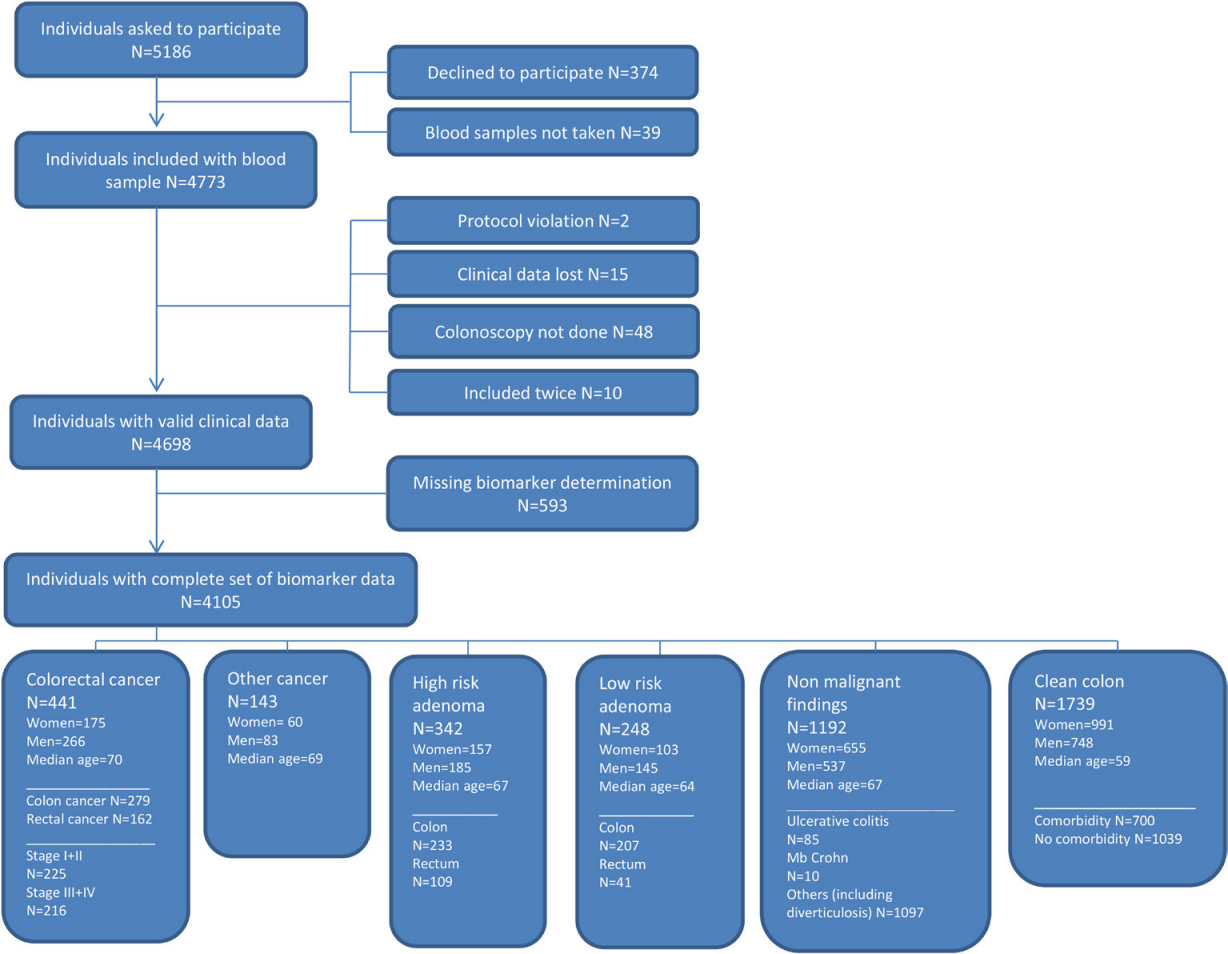
Flowchart showing the inclusion of individuals in the study

A total of 4,105 individuals referred to diagnostic colonoscopy were included in the study comprising 441 individuals with CRC (279 with colon cancer and 162 with rectal cancer), 143 with primary cancer of non-colorectal origin and 342 with high risk adenomas (Figure 1). The population included 1,964 men and 2,141 women with median age of 64 (18–95).

**Figure 2:**
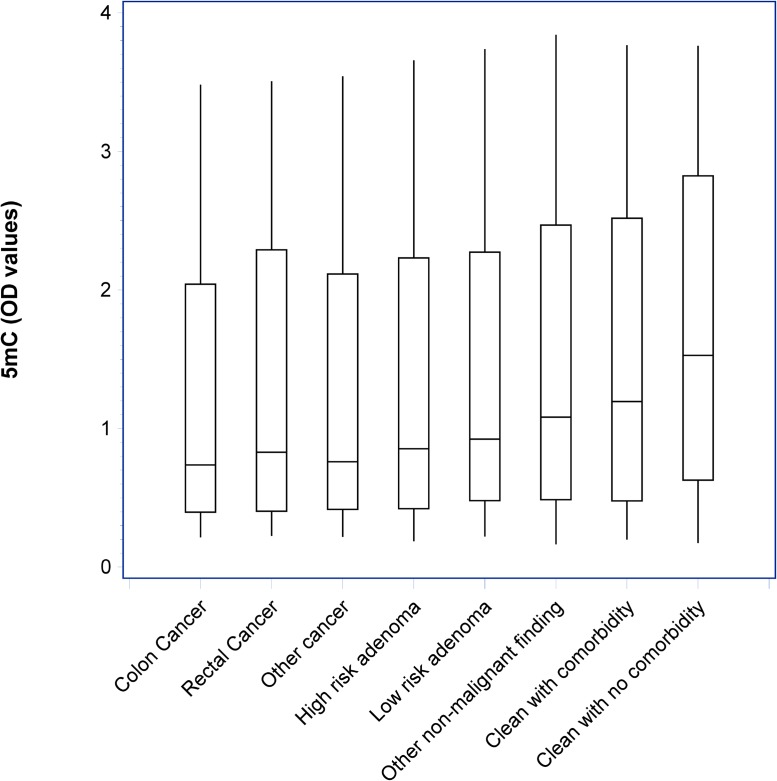
Box-plots with median, 1st and 3rd quartile showing the distribution of optical density (OD) values of circulating cell-free nucleosomes containing 5-methylcytosine DNA (5 mC) according to diagnostic groups A significant trend (*p* < 0.001) of decreased levels in individuals with CRC, other cancers and colorectal adenomas are shown compared with individuals with other non-malignant findings and clean colorectum.

Figure 2 shows the distribution of optical density (OD) measurements of 5 mC according to diagnostic groups. A significant trend (*p* < 0.001) of decreased levels in individuals with CRC, other cancers and colorectal adenomas are shown compared with individuals with other non-malignant findings and clean colorectum.

**Figure 3:**
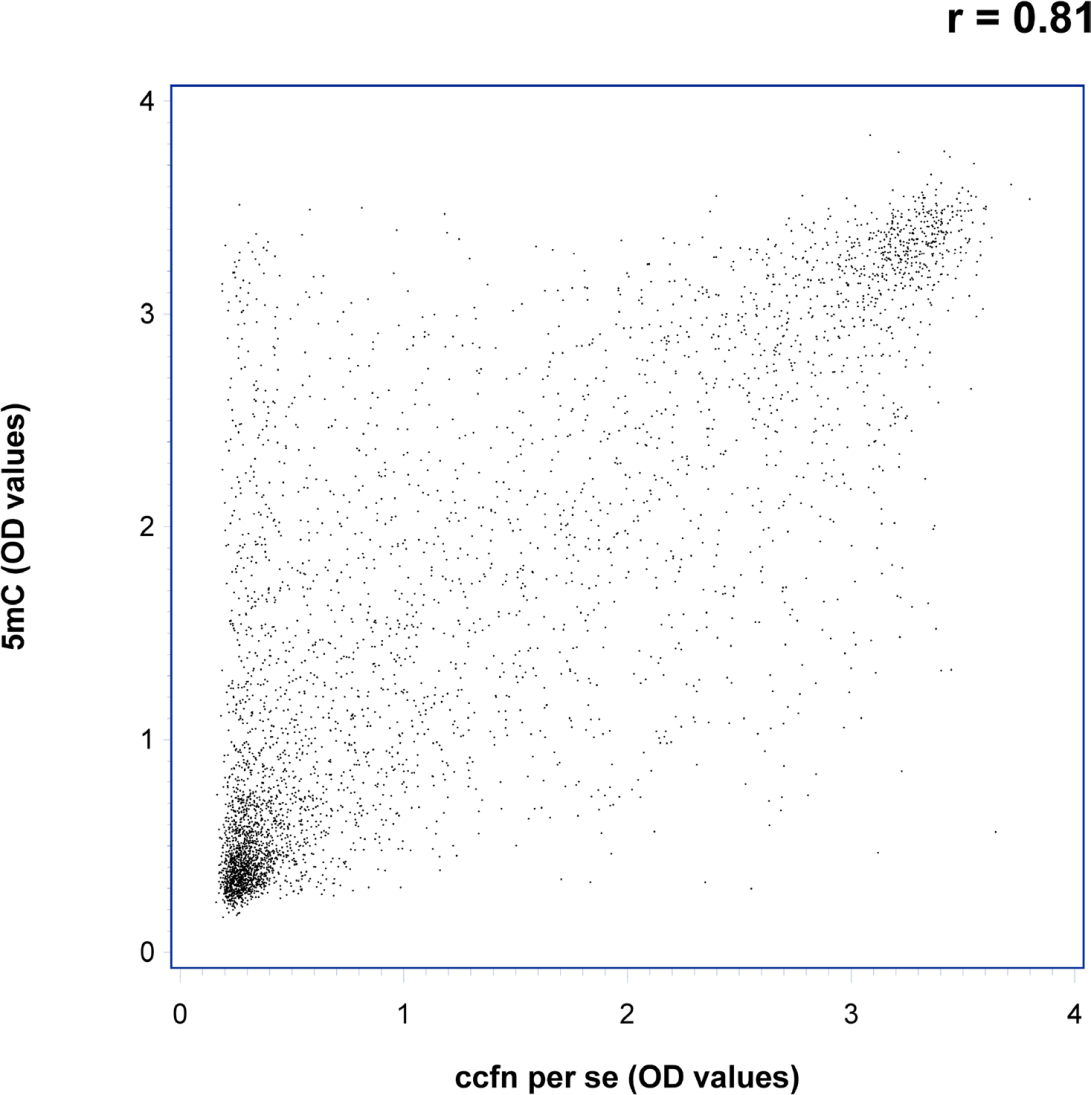
Scatter plot showing optical density (OD) values of circulating cell-free nucleosomes containing 5-methylcytosine DNA (5 mC) vs. total circulating cell-free nucleosomes per se (ccfn per se) illustrating the correlation between the two measurements, r = 0.81 (*p* < 0.001)

Spearman rank correlations were used to assess the association between the ccfn. Correlation coefficients were found to be > 0.5 except for H3K9Me3 where the correlation coefficients with the other ccfn were > 0.3 (data not shown). Figure 3 shows a scatter plot of OD-measurements of 5 mC vs. ccfn per se illustrating the correlation between the two measurements with a correlation coefficient of 0.81 (*p* < 0.001).

Results of the assessment of influence of disease stage, gender and comorbidity on levels of ccfn and CEA are presented in Table 1. An independence of disease stage (CRC stage I–IV) was shown as comparisons of levels of the ccfn in the four disease groups did not reach significance. As expected, levels of CEA were significantly stage dependent with higher levels associated to higher stage. The level of H3S10PO4 was the only ccfn that showed an association to gender with significantly lower levels in women compared to men; however, all ccfn had R^2^ < 0.02 (Table 1). Regarding influence of comorbidity, levels of 5 mC, H4PanAc, H3K9Ac, H3S10PO4, H3K36Me3, EZH2, HMGB1 and CEA (lung disease), H3K9Me3 and pH2AX (cardiovascular disease), and H3K9Me3 (diabetes I/II) were significantly lower in individuals with comorbidity compared to levels in individuals with no comorbidity and clean colorectum; R^2^ < 0.05 (Table 1). In the comparison of individuals with rheumatic disease to individuals with no comorbidity and clean colorectum, significance was not reached for levels of any ccfn or CEA (Table 1).

**Table 1:** R^2^ and p-values of the difference in serum levels of modified circulating cell-free nucleosomes and carcinoembryonic antigen (CEA) between individuals with stage I–IV colorectal cancer (CRC), between individuals with comorbidity (cardiovascular disease, diabetes I/II, lung disease and rheumatic disease) and healthy individuals, and between gender and age in healthy individuals

	CRC		Co-morbidity								Demographics			
	CRC stages		Cardiovascular		Diabetes I/II		Lung		Rheumatic		Age		Gender	
	R^2^	*p*-value	R^2^	*p*-value	R^2^	*p*-value	R^2^	*p*-value	R^2^	*p*-value	R^2^	*p*-value	R^2^	*p*-value
**Parameter_a_**														
**ccfn per se**	0.0060	0.4517	0.0260	0.0503	0.0238	0.9370	0.0248	0.1846	0.0238	0.8862	0.0071	0.0067	0.0010	0.2980
**5mC**	0.0040	0.6239	0.0320	0.3676	0.0315	0.8800	0.0348	0.0153	0.0318	0.4676	0.0151	< 0001	0.0015	0.2122
**H3K9Me3**	0.0148	0.0900	0.0155	0.0325	0.0155	0.0315	0.0141	0.1359	0.0130	0.6581	0.0083	0.0032	0.0016	0.2001
**H3K9Ac**	0.0060	0.4506	0.0190	0.5074	0.0191	0.4145	0.0215	0.0273	0.0188	0.9918	0.0062	0.0114	0.0007	0.4048
**H3S10PO4**	0.0060	0.4542	0.0172	0.1177	0.0158	0.9394	0.0193	0.0124	0.0159	0.6600	0.0059	0.0136	0.0039	0.0431
**H3K36Me3**	0.0048	0.5541	0.0127	0.4804	0.0124	0.9788	0.0153	0.0244	0.0124	0.9948	0.0038	0.0469	0.0024	0.1152
**H4K20Me3**	0.0061	0.4416	0.0160	0.0548	0.0146	0.2867	0.0148	0.2263	0.0147	0.2510	0.0075	0.0053	0.0024	0.1148
**H4PanAc**	0.0055	0.4931	0.0103	0.2554	0.0101	0.3364	0.0123	0.0284	0.0097	0.7029	0.0034	0.0603	0.0014	0.2211
**pH2AX**	0.0052	0.5133	0.0246	0.0155	0.0216	0.4350	0.0233	0.0613	0.0213	0.7535	0.0056	0.0160	0.0029	0.0815
**H2AZ**	0.0046	0.5743	0.0201	0.1532	0.0190	0.6158	0.0207	0.0730	0.0190	0.6946	0.0075	0.0052	0.0036	0.0543
**HMGB1**	0.0119	0.1552	0.0209	0.0972	0.0194	0.9220	0.0217	0.0453	0.0195	0.6188	0.0072	0.0062	0.0015	0.2174
**EZH2**	0.0067	0.3993	0.0209	0.0626	0.0194	0.3543	0.0235	0.0047	0.0195	0.3337	0.0062	0.0109	0.0034	0.0605
**CEA**	0.2046	< 0001	0.0108	0.3492	0.0105	0.5051	0.0207	< 0001	0.0109	0.2928	0.0053	0.0193	0.0004	0.5461

_a_ ccfn: circulating cell-free nucleosomes, 5mC: ccfn containing 5-methylcytosine DNA, H3K9Me3: histone H3 tri-methylated at lysine 9, H3K9Ac: histone H3 acetylated at lysine 9, H3S10PO4: histone H3 phosphorylated at serine 10, H3K36Me3: histone H3 tri-methylated at lysine 36, H4K20Me3: histone H4 tri-methylated at lysine 20, H4PanAc: pan-acetylated H4, pH2AX: histone variant H2AX phosphorylated at serine 139, H2AZ: nucleosome variant H2AZ, HMGB1: nucleosome adducted with HMGB1, EZH2: nucleosome adducted with EZH2.

A weak dependence of levels of ccfn on age was shown with decreasing levels at increasing age (R^2^ < 0.02)

Results from the univariate analysis of the ccfn and CEA regarding the discrimination of individuals with CRC (endpoint 1), individuals with CRC and high-risk adenomas (endpoint 2), individuals with cancer (endpoint 3), individuals with high risk adenomas (endpoint 4) and individuals with other cancers excluding CRC (endpoint 5) are shown in Table 2. For endpoint 1, the area under the receiver operating characteristic (ROC) curve (AUC_ROC_) was in the range of 0.525–0.576; results for H3K36Me3 and H4K20Me2 were not significant, but significance was reached for the remaining ccfn. Regarding endpoint 2 and 3, the AUC_ROC_ was found to be in the range of 0.526–0.574; results for all ccfn were significant. AUC_ROC_ was 0.533–0.584 for endpoint 4; results for all ccfn were significant except ccfn per se. Results for endpoint 5 showed insignificant result for the majority of ccfn with the exception of ccfn per se, 5 mC, H3K9Me3 and pH2AX; AUC_ROC_ was 0.508–0.564.

**Table 2:** Area under the receiver operating characteristic (ROC) curve (AUC_ROC_) and p-values of univariate logistic regression analysis of endpoint 1–5_a_ based on serum levels of circulating cell-free nucleosomes and carcinoembryonic antigen (CEA)

	Endpoint_a_									
	1		2		3		4		5	
	AUC_ROC_	*P*-value	AUC_ROC_	*P*-value	AUC_ROC_	*P*-value	AUC_ROC_	*P*-value	AUC_ROC_	*P*-value
**Parameter_b_**										
**ccfn per se**	0.559	< 0001	0.549	< 0001	0.557	< 0001	0.533	0.0911	0.552	0.0480
**5mC**	0.574	< 0001	0.574	< 0001	0.571	< 0001	0.563	< 0001	0.564	0.0112
**H3K9Me3**	0.539	0.0050	0.546	< 0001	0.541	0.0007	0.551	0.0031	0.554	0.0261
**H3K9Ac**	0.567	< 0001	0.565	< 0001	0.560	< 0001	0.556	0.0009	0.536	0.1143
**H3S10PO4**	0.547	0.0025	0.543	< 0001	0.539	0.0021	0.534	0.0089	0.520	0.2871
**H3K36Me3**	0.525	0.1010	0.542	0.0004	0.526	0.0434	0.557	0.0008	0.532	0.1691
**H4K20Me3**	0.526	0.0707	0.546	0.0003	0.531	0.0271	0.563	0.0011	0.542	0.1455
**H4PanAc**	0.544	0.0110	0.554	< 0001	0.546	0.0048	0.561	0.0021	0.543	0.1554
**pH2AX**	0.576	< 0001	0.574	< 0001	0.573	< 0001	0.562	0.0002	0.564	0.0487
**H2AZ**	0.540	0.0115	0.563	< 0001	0.536	0.0088	0.584	< 0001	0.530	0.2821
**HMGB1**	0.558	< 0001	0.561	< 0001	0.556	< 0001	0.557	0.0015	0.552	0.0602
**EZH2**	0.540	0.0096	0.542	0.0002	0.531	0.0095	0.542	0.0079	0.508	0.3867
**CEA**	0.662	< 0001	0.606	< 0001	0.668	< 0001	0.530	0.1862	0.688	< 0001

_a_ Endpoint 1: Individuals with colorectal cancer (CRC) vs. individuals with non-malignant findings; Endpoint 2: Individuals with CRC and high risk adenomas vs. individuals with non-malignant findings excluding individuals with high risk adenomas; Endpoint 3: Individuals with cancer vs. individuals with non-malignant findings; Endpoint 4: Individuals with high risk adenomas vs. individuals with non-malignant findings excluding individuals with high risk adenomas; Endpoint 5: Individuals with cancer excluding CRC vs. individuals with non-malignant findings.

_b_ ccfn: circulating cell-free nucleosomes, 5mC: ccfn containing 5-methylcytosine DNA, H3K9Me3: histone H3 tri-methylated at lysine 9, H3K9Ac: histone H3 acetylated at lysine 9, H3S10PO4: histone H3 phosphorylated at serine 10, H3K36Me3: histone H3 tri-methylated at lysine 36, H4K20Me3: histone H4 tri-methylated at lysine 20, H4PanAc: pan-acetylated H4, pH2AX: histone variant H2AX phosphorylated at serine 139, H2AZ: nucleosome variant H2AZ, HMGB1: nucleosome adducted with HMGB1, EZH2: nucleosome adducted with EZH2.

Results from the predictor models (AUC_ROC_ with associated *p*-value and sensitivities at specificities 0.70, 0.80, and 0.90) are presented in Table 3. The ccfn with independent significance and thus included in the predictor model 1–5 (based on endpoint 1–5, respectively) was 5 mC. The discrimination of individuals with CRC from individuals with non-malignant findings (endpoint 1) yielded AUC_ROC_ = 0.736 (sensitivity = 0.37 at specificity = 0.90) when applying predictor 1. Similar AUC_ROC_ was shown in the discrimination of individuals with cancer (CRC and other cancers) (endpoint 3) and individuals with other cancers (excluding CRC) (endpoint 5); AUC_ROC_= 0.736 (sensitivity = 0.37 at specificity = 0.90) and AUC_ROC_= 0.741 (sensitivity 0.39 at specificity 0.90), respectively. In comparison, the discrimination of individuals with high risk adenomas (endpoint 4) and individuals with CRC and high risk adenomas (endpoint 2) from individuals with non-malignant findings were lower; AUC_ROC_ = 0.646 (sensitivity = 0.21 at specificity = 0.90) and AUC_ROC_= 0.691 (sensitivity = 0.28 at specificity = 0.90), respectively.

**Table 3:** Assessment of predictor models and subgroup-predictor models: Area under the receiver operating characteristic (ROC) curve (AUC_ROC_) and sensitivity at specificities 0.7, 0.8 and 0.9

Sensitivity		Specificity		
		0.7	0.9	0.8
		Sensitivity	Sensitivity	Sensitivity
**Predictor model**	**AUC_ROC_**			
**Endpoint 1 (A)**	**0.736**	0.615	0.510	0.370
**Endpoint 2 (B)**	**0.691**	0.571	0.420	0.284
**Endpoint 3 (C)**	**0.736**	0.622	0.524	0.368
**Endpoint 4 (D)**	**0.646**	0.480	0.360	0.205
**Endpoint 5 (E)**	**0.741**	0.636	0.566	0.385
**Endpoint 1/stage I-II (Fa)**	**0.716**	0.596	0.484	0.329
**Endpoint 1/stage III-IV (Fb)**	**0.755**	0.633	0.535	0.409
**Endpoint 1/stage I-III (Fc)**	**0.711**	0.566	0.451	0.310
**Endpoint 1 vs clean colorectum (G)**	**0.840**	0.810	0.737	0.565
**Endpoint 1/stage I-II vs clean colorectum (Ha)**	**0.832**	0.791	0.720	0.551
**Endpoint 1/stage III-IV vs clean colorectum (Hb)**	**0.858**	0.828	0.753	0.577
**Endpoint 2 vs clean colorectum (I)**	**0.813**	0.793	0.693	0.506

**Figure 4:**
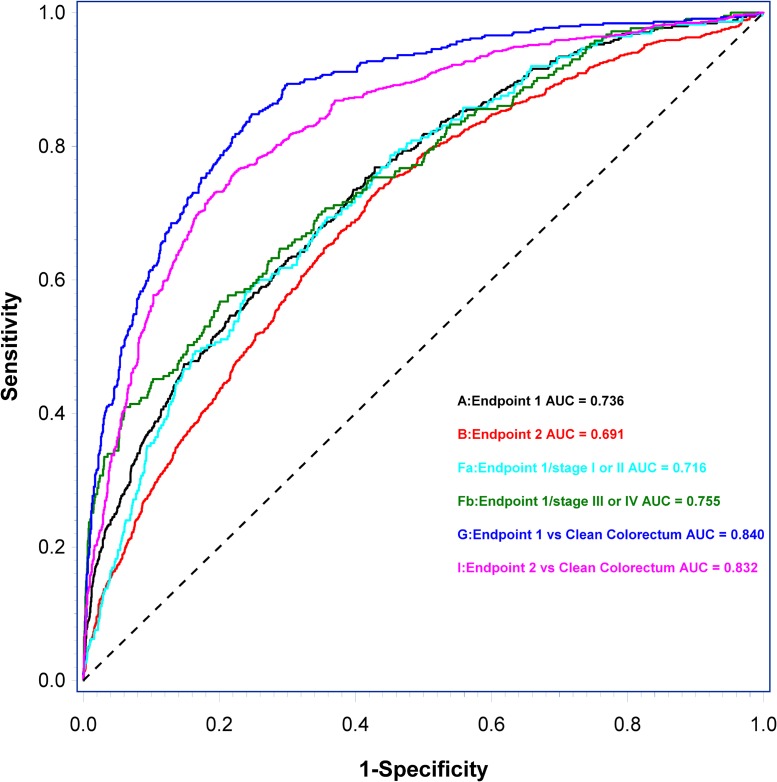
Receiver operating characteristic (ROC) curves and area under the ROC curve (AUC_ROC_) of predictor models and subgroup-predictor models based on the discrimination of endpoint 1 (**A**), endpoint 2 (**B**), the discrimination of early stage CRC (Fa) and late stage CRC (Fb) from individuals with non-malignant findings, the discrimination of individuals with CRC (G) from individuals with clean colorectum, and the discrimination of individuals with CRC and high risk adenomas (I) from individuals with clean colorectum. Letters in parenthesis refer to results from Predictor models listed in Table 3.

Letters in parenthesis refer to ROC curves shown in Figure 4. Circulating cell-free nucleosomes containing 5-methylcytosine DNA and CEA were included as explanatory variables along with age and gender in all five predictor models. Endpoint 1: Individuals with colorectal cancer (CRC) vs. individuals with non-malignant findings; Endpoint 2: Individuals with CRC and high risk adenomas vs. individuals with non-malignant findings excluding individuals with high risk adenomas; Endpoint 3: Individuals with cancer vs. individuals with non-malignant findings; Endpoint 4: Individuals with high risk adenomas vs. individuals with non-malignant findings excluding individuals with high risk adenomas; Endpoint 5: Individuals with cancer excluding CRC vs. individuals with non-malignant findings.

To further explore the performance of the predictor models based on serum levels of ccfn, CEA, age and gender, subgroup-predictor models were developed. Compared to endpoint 1, subgroup-predictor model based on the discrimination of individuals with CRC from individuals with clean colorectum showed an improved AUC_ROC_ and associated sensitivity (AUC_ROC_ = 0.840, sensitivity = 0.57 at specificity = 0.90). Subgroup-predictor model based on the discrimination of individuals with early stage CRC (stage I or II) and late stage CRC (stage III or IV) from individuals with clean colorectum resulted in a difference in AUC_ROC_ of 0.026 in favor of late stage CRC. Eliminating CEA from the subgroup-predictor models resulted in a difference of 0.026 in favor of early stage CRC (data not shown).

Finally, a subgroup predictor model based on the discrimination of individuals with CRC stage I + II + III from individuals with non-malignant findings, thus eliminating the influence of metastatic disease, showed AUC_ROC_ = 0.711, sensitivity = 0.31 at specificity = 0.90.

Figure 4 shows ROC curves and AUC_ROC_ of predictor model 1 and 2 and subgroup-predictor models based on the discrimination of early and late stage CRC from individuals with non-malignant findings, the discrimination of individuals with CRC from individuals with clean colorectum and the discrimination of individuals with CRC and high risk adenomas from individuals with clean colorectum.

## DISCUSSION

Assessed individually, the determined ccfn have a weak ability to discriminate individuals with high risk adenomas and cancer including CRC from individuals with non-malignant findings (AUC_ROC_ = 0.508–0.584). However, the performance was substantially improved when predictor models based on endpoint 1–5 were constructed combining levels of ccfn determinations (5 mC) with levels of CEA, age and gender. The performance in discrimination of individuals with CRC from individuals with non-malignant findings yielded AUC_ROC_ of 0.736. This is in line with previous research showing association between specific ccfn alterations and CRC [12, 19, 22], and the improved performance by combination of ccfn as biomarker for cancer [17, 19].

The performance of the predictor models in discrimination of individuals with high risk adenomas and CRC (endpoint 2) and individuals with high risk adenomas exclusively (endpoint 4) obtained AUC_ROC_ = 0.691 and AUC_ROC_ = 0.646, respectively. The inferior AUC_ROC_ compared with the performance in discrimination of individuals with CRC may be explained by the non-invasive nature of adenomas compared to carcinomas, which could cause a reduction in release of altered ccfn into the circulation. Supporting this speculation, previous research has showed that the profile of epigenetic alterations in tissue from colorectal adenomas and CRC are similar, and the expression of some alterations is higher in tissue from CRC compared to tissue from adenomas [23, 24].

In comparison to the performance in discrimination of individuals with CRC, the performance in discrimination of individuals with cancer including CRC (endpoint 3) and individuals with other cancers excluding CRC (endpoint 5) obtained similar results, AUC_ROC_ = 0.736 and AUC_ROC_ = 0.741, respectively. This may indicate that ccfn represent epigenetic changes associated with carcinogenesis in general and not only carcinogenesis of CRC. However, the findings regarding discrimination of individuals with other cancers are based on a relatively small number of individuals, and further studies are needed to draw a final conclusion.

The performance of the predictor models was further improved in discrimination of individuals with CRC from individuals with clean colorectum which yielded AUC_ROC_ of 0.840 (sensitivity 0.57 at specificity 0.90). This discrimination was performed to provide an indication of the maximum potential performance of the predictor models in a screening setting, as the performance based on endpoint 1–5 might be underestimated due to the design of the study with the inclusion of a symptomatic population. Thus, the individuals of the control group are symptomatic.

The progress of CRC does not influence levels of ccfn as variations in levels of ccfn between the groups of individuals of the four stages of CRC were insignificant (Table 1). The performance of predictor model 1 in discrimination of individuals with early stage CRC compared to discrimination of individuals with late stage CRC is small, and the difference in AUC_ROC_ in favor of late stage disease reverts to favor early stage disease when CEA is eliminated from the predictor model. Furthermore, similar performance of the predictor model was obtained when metastatic CRC (stage IV) was eliminated from the discrimination. Altogether, this indicates that levels of ccfn are independent of progression of CRC. This might be explained by epigenetic changes playing a qualitative role in carcinogenesis. Hence, it is speculated that the amount of DNA and histone changes in the nucleosomes is persistent from the initial neoplastic transformation to the advanced cancer stage making ccfn ideal as a biomarker for early detection of CRC. The independence of disease stage would be an advantageous quality as potential blood-based screening-biomarker for CRC.

Exclusively, 5 mC was the only ccfn included in the predictor models. This may be explained by the relatively strong correlations observed between the ccfn (Spearman rank correlation) indicating a close and complex interaction between the different histone modifications, variants and nucleosomes containing methylated DNA. The effect of a single nucleosomic modification on the development of CRC may be linked to the effect of numerous modifications in a dynamic complex of various epigenetic regulatory mechanisms [25].

In this study, the blood samples were collected before colonoscopy but after bowel preparation which potentially could affect levels of biomarkers and thereby might induce bias. However, it has been shown that levels of ccfn are not influenced by bowel preparation [26] which supports the potential use of ccfn in screening for CRC even further.

Levels of ccfn decline with increasing age although the overall influence of age is minor as the proportion of the explained variance is very low. In general, the effect of aging on epigenomic regulation of DNA is complex and involves interaction between multiple regulatory proteins and pathways. During aging, a general loss of heterochromatin has been shown which causes a loss of repressive histone marks and methylated DNA [27]. This supports the present findings of decreasing levels of ccfn with increasing age. The effect of gender on levels of ccfn was concluded to be of limited importance since only levels of H3S10PO4 showed difference between genders and all ccfn obtain R^2^-values < 0.02. Lung disease, cardiovascular disease and diabetes I/II were found to affect levels of some ccfn, but again the influence was found to be minor.

The levels of ccfn were measured in output from ELISA as OD values. However, the assay does not distinguish between the numbers of times a specific alteration appears in a single nucleosome. Hence, the OD output from the ELISA detection is not equivalent to a concentration of ccfn, but is an expression of the amount of altered epigenetic control of gene expression in serum samples.

The Sept9 DNA methylation assay was the first blood-based CRC screening test to be approved for use in an average risk population [28] with a reported sensitivity of 0.48 at specificity 0.915 [29] in an asymptomatic population. The detection rates are correlated to the progression of CRC as advanced disease leads to increased detection rates, and the detection rates for high risk adenomas are low. The performance of the Sept9 DNA methylation assay has been shown to be influenced by age and comorbidities such as diabetes [30]. A direct comparison between the levels of ccfn as blood-based biomarker for early detection of CRC found in this study with the performance of the Sept9 DNA methylation assay is not possible due to differences in study-design. However, the independence of progression of CRC, the minor influence of co-morbidity and age, the relatively high performance in discrimination of individuals with high risk adenomas and the high performance in discrimination of individuals with CRC from individuals with clean colorectum found in this study, may indicate that levels of ccfn are competitive to Sept9 DNA methylation assay as blood-based biomarkers for early detection of CRC. However, further studies based on asymptomatic populations are needed to confirm the results of this study.

The epigenetic regulation of DNA composes a complex interaction between different modifications leading to diverse patterns of gene expression emerging from the same genome [25]. The ccfn evaluated in this study are all associated with CRC, but are not separate events in the epigenetic regulation of the genome during carcinogenesis, reflecting only a tip of the iceberg of the entire epigenetic alterations leading to CRC. This explains the finding of relatively strong correlations between the ccfn.

In conclusion, the performance of the designed predictor models is potentially promising in early detection of CRC. Addition of biomarkers of different quality, for example protein biomarkers, to the present predictor-model based on epigenetic modifications might improve the performance even further, making the biomarker-combination potentially competitive to FIT and thereby beneficial supplement in a screening program. Another future aspect is the possible supplement of the predictor-models to FIT in order to potentially improve performance of current screening-programs. However, further studies are needed to support these proposals.

## MATERIALS AND METHODS

The study was a part of the Endoscopy II Project, which has been approved by the Ethics Committee of the Capital Region of Denmark (H-3-2009-110) and the Danish Data Protection Agency (2007-58-0015) and performed according to the Helsinki II Declaration.

Individuals scheduled for first time ever diagnostic colonoscopy due to symptoms attributable to CRC at seven hospitals in Denmark (Bispebjerg, Herning, Hillerød, Horsens, Hvidovre, Randers, and Aarhus hospitals) were included from 1 May 2010 to 30 November 2012. Inclusion criteria were age ≥ 18, symptoms attributable to CRC and signed informed consent. Exclusion criteria were previous malignant disease, previous large bowel adenoma, member of a Hereditary Non-Polyposis Colorectal Cancer or Familiar Adenomatosis Polyposis family and surgical intervention within 3 months before inclusion. Furthermore, individuals unable to understand the written study-information were excluded from the study. On the day of the colonoscopy an informed consent was signed by the participating individual, and personal data (age, gender, comorbidity (cardiovascular disease, lung disease, rheumatic disease, and diabetes I/II)) was recorded by a dedicated research nurse. Blood samples for serum were collected before colonoscopy according to a validated Standard Operating Procedure and subsequently centrifuged at 3,000 X G for 10 minutes at 21°C. The supernatant serum was transferred to separate freezing tubes leaving 0.5 cm of serum untouched above the buffy-coat to avoid contamination from white cells and platelets. Serum samples were labelled with unique barcodes to ensure subsequent identification (FreezerWorks^®^), and all samples were stored at −80°C under 24/7 electronic surveillance.

Findings at colonoscopy including pathological diagnosis were registered in a database in addition to the obtained personal data. After final data compilation a rigorous audit of the database was performed to ensure the validity, and the database was locked.

The endpoints considered were:

Endpoint 1: Individuals with CRC (all stages), Endpoint 2: Individuals with CRC and high risk adenomas, Endpoint 3: Individuals with cancer (CRC and cancer of non-colorectal origin). Endpoint 4: Individuals with high risk adenomas, Endpoint 5: Individuals with other cancers (excluding CRC).

The control group for endpoint 1, 3 and 5 was individuals with non-malignant findings including low and high risk adenomas. The control group for endpoint 2 and 4 was individuals with non-malignant findings including low risk adenomas and excluding high risk adenomas.

The criteria for high risk adenoma were: ≥ 1 cm in size or ≥ 3 lesions or villous component or high grade dysplasia [31].

The panel of 12 ccfn assays were constructed based on literature searches on published results for epigenetic modifications associated with carcinogenesis of CRC as well as previous experiences of ccfn modifications and adducts associated with CRC.

Ccfn and CEA levels in the serum samples were determined by Belgian Volition SPRL, Isnes, Belgium using Nu.Q^TM^ ELISA platforms as previously described [12]. In brief, ccfn were captured onto an ELISA plate using an anti-nucleosome antibody and the level of nucleosomes containing a particular epigenetic feature was quantified by binding of a separate detection antibody directed to bind to the epigenetic feature of the nucleosomes.

Absolute mass or other SI units of ccfn have not yet been defined, and no International Standard preparations, absolute or relative, have been reported. Therefore, the performed measurements of levels of ccfn and CEA were expressed in the output from the ELISA detection as OD. All ELISA measurements on each serum sample were performed in duplicate and results used for the statistical analysis were expressed as the mean of the duplicate measurement.

Only results from individuals with a complete set of biomarker determinations (5 mC, H3K9Me3, H3K9Ac, H3S10PO4, H3K36Me3, H4K20Me3, H4PanAc, pH2AX, H2AZ, HMGB1, EZH2, ccfn per se and CEA) were used for statistical analyses. The flow chart (Figure 1) presents an overview of individuals recruited and included or excluded in the study. Missing biomarker determinations were due to lack of sample material and were missing at random.

### Statistics

Comparison of ccfn levels between CRC stages, co-morbidities, gender and age were done using linear modelling with the ccfn levels log transformed. Analysis of co-morbidities, gender and age were restricted to individuals with a clean colorectum result. In addition, the analysis of co-morbidities was adjusted for age and gender. The results of these analyses are presented by the R^2^ value (reflecting proportion of the total variation explained by the model) and the *p*-value.

All endpoints considered are binary as described above. Logistic regression analysis with the endpoints as the dependent variable and the biomarkers as explanatory variables has been done. Age and gender have also been included as explanatory covariates. Initial analyses were done for each explanatory variable as a univariate analysis, and then multivariate analysis was performed combining all explanatory variables as well as age and gender, reducing the model to only include statistically significant ccfn. The final model was developed using 10-fold cross validation with backwards selection using the Akaike information criterion. The results are presented by the ROC curves with AUC_ROC_ as a measure of discrimination and the sensitivities at pre-specified specificities (70, 80, and 90%). For each endpoint, a predictor, i.e. a linear combination of the significant explanatory covariates, has been established; exclusively, 5 mC and CEA were included as explanatory variables along with age and gender in all five predictor models. Subgroup analyses restricted to early stage CRC (stage I or II), late stage CRC (stage III or IV), and restricting the controls to individuals with clean colorectum have been performed. *P*-values less than 5% are considered significant. Database management and calculations have been done using SAS (v9.4, SAS Institute, Cary, N.C., USA) and R (Frank E Harrell Jr (2016). Rms: Regression Modeling Strategies. R package version 4.4-2 http://CRAN.R-project.org/package=rms).

## Appendix

Hans Jørgen Nielsen^1^, Ib Jarle Christensen^1^, Lars Nannestad Jørgensen^2^, Mogens R Madsen^3^, Jesper Vilandt^4^, Thore Hillig^5^, Michael Klærke^6^, Knud T Nielsen^7^, Søren Laurberg^8^, Nils Brünner^9^. Department of Surgical Gastroenterology at Hvidovre^1^, Bispebjerg^2^, Herning^3^, Hillerød^4^, Horsens^6^, Randers^7^ and Aarhus^8^ Hospitals, Denmark; Department of Clinical Biochemistry, Hillerød5 Hospital, Denmark; Department of Veterinary Disease Biology^9^, Faculty of Health and Medical Sciences, University of Copenhagen, Denmark.

## Abbreviations

CRC: colorectal cancer
FIT: fecal immunochemical test
ccfn: circulating cell-free nucleosomes
ELISA: enzyme-linked immunosorbent assay
5 mC: ccfn containing 5-methylcytosine DNA
H3K9Me3: ccfn containing histone H3 tri-methylated at lysine 9
H3K9Ac: ccfn containing histone H3 acetylated at lysine 9
H3S10PO4: ccfn containing histone H3 phosphorylated at serine 10
H3K36Me3: ccfn containing histone H3 tri-methylated at lysine 36
H4K20Me3: ccfn containing histone H4 tri-methylated at lysine 20
H4PanAc: ccfn containing pan-acetylated H4
pH2AX: ccfn containing histone variant H2AX phosphorylated at serine 139
H2AZ: ccfn containing nucleosome variant H2AZ
HMGB1: ccfn containing nucleosome adducted with HMGB1
EZH2: ccfn containing nucleosome adducted EZH2
CEA: carcinoembryonic antigen
OD: optical density
ROC: receiver operating characteristic
AUC_ROC_: area under the ROC curve
OD: optical density
